# Preparation of Laser-Ablated Ag Nanoparticle–MMT Clay-Based Beeswax Antibiofilm Coating

**DOI:** 10.3390/antibiotics12020194

**Published:** 2023-01-17

**Authors:** Syed Imdadul Hossain, Diellza Bajrami, Maria Chiara Sportelli, Rosaria Anna Picca, Annalisa Volpe, Caterina Gaudiuso, Antonio Ancona, Luigi Gentile, Gerardo Palazzo, Nicoletta Ditaranto, Boris Mizaikoff, Nicola Cioffi

**Affiliations:** 1 Chemistry Department, University of Bari “Aldo Moro”, Via E. Orabona 4, 70126 Bari, Italy; 2 CSGI (Center for Colloid and Surface Science) c/o, Department of Chemistry, Via Orabona 4, 70125 Bari, Italy; 3 Institute of Analytical and Bioanalytical Chemistry, Ulm University, Albert Einstein-Allee 11, 89081 Ulm, Germany; 4 Physics Department “M. Merlin”, University of Bari “Aldo Moro”, Via E. Orabona 4, 70126 Bari, Italy; 5 Politecnico of Bari, Via E. Orabona 4, 70126 Bari, Italy; 6 Physics Department “M. Merlin”, IFN–CNR (Istituto di Fotonica e Nanotecnologie–Consiglio Nazionale delle Ricerche), Via Amendola 173, 70126 Bari, Italy; 7 Hahn-Schickard Institute for Microanalysis Systems, Sedanstrasse 14, 89077 Ulm, Germany

**Keywords:** antimicrobial, antibiofilms, silver, montmorillonite, beeswax, coating

## Abstract

Unlike other antimicrobial agents, Ag-based composites are stable and currently widely used as broad spectral additives, fighting microbial biofilms and other biological threats. The goal of the present study is to develop a green, multifunctional, and robust antibiofilm water-insoluble coating, inhibiting histamine-producing *Lentilactobacillus parabuchneri* biofilms. Herein, laser-ablated Ag NPs (L-Ag NPs) were incorporated into and onto a montmorillonite (MMT) surface layer with a simple wet chemical method, provided that the electrostatic interaction between L-Ag NPs and MMT clay led to the formation of L-Ag/MMT nanoantimicrobials (NAMs). The use of MMT support can facilitate handling Ag NPs in industrial applications. The Ag/MMT composite was characterized with transmission electron microscopy (TEM) and scanning electron microscopy (SEM), which confirmed the entrapment of L-Ag NPs into MMT clay. The surface chemical composition was assessed with X-ray photoelectron spectroscopy, proving that Ag NPs were in contact with and deposited onto the surface of MMT. The characteristic L-Ag/MMT band was investigated with UV–vis spectroscopy. Following that, the L-Ag/MMT composite was embedded into a biosafe water-insoluble beeswax agent with a spin coating technique. The antimicrobial ion release kinetic profile of the L-Ag/MMT/beeswax coating through an electrothermal atomic absorption spectroscopy (ETAAS) study supported the controlled release of Ag ions, reaching a plateau at 420 ± 80 nM, which is safe from the point of view of Ag toxicity. Microbial biofilm growth inhibition was assessed with real-time in situ Fourier transform infrared attenuated total reflection spectroscopy (FTIR-ATR) in a flow cell assembly over 32 h. The study was further supported by optical density (OD) measurements and SEM on bacteria incubated in the presence of the L-Ag/MMT/beeswax coating.

## 1. Introduction

Antimicrobial resistance (AMR) is a global threat facing humanity. The European Centre for Disease Prevention and Control (ECDC) reported that AMR is a growing concern to public health [1]. The significant impact of AMR on the economy, health, and society includes human disability, severe illness, and longer hospital stays, which require more expensive medicines. The success of medicine in treating infection and major surgery would be at high risk without the support of effective nanoantimicrobials (NAMs) because, on the one hand, currently available and newly developed antibiotics are not enough to race against AMR; on the other hand, bacteria produce colonies on medical devices and food packaging. Viruses, bacteria, fungi, and parasites have the ability to transfer genes, changing their mechanism of action over time, surviving harsh environments, and becoming so strong that they render antibiotics and antimicrobials ineffective. AMR organisms may be found in people, animals, food, plants, soil, and air [2]. Urgent action and plans are imperative for the sustainable development of antimicrobial materials. A lack of access to effective antimicrobials remains a major issue. For those reasons, extensive research focus should be devoted to developing novel antimicrobial agents. However, challenges still remain, as antimicrobial resistance is continuously occurring globally, and the overuse of antimicrobial agents poses the possibility of increasing antimicrobial resistance. Therefore, the use and concept of AM agents should be optimized. The aim is to design particular synergistic AM agents with the multifunctional activities, ecofriendly material, optimized dose, concentration ion release kinetics, shape, and size of NAMs. Top priority should be given to the development of potential AM agents that can inhibit and eradicate biofilms, which are particularly detrimental to the hospital and industrial sectors, for instance, in food packaging applications. Lactic acid bacteria such as *Lentilactobacillus parabuchneri* are associated with the production of histamine in cheese [3,4,5]. Biogenic amine (BA) histamine is toxic and responsible for food poisoning. The ingestion of high levels of histamine may cause rashes, headaches, a hypertensive crisis, and gastrointestinal and respiratory problems. Considering its toxicological effects, histamine-forming *L. parabuchneri* strain poses a major risk in the food industry. *L. parabuchneri* strain has the undesirable tendency and strong ability to form biofilms on dairy industry equipment surfaces, further increasing the food toxicity level. *L. parabuchneri* biofilms often develop resistance against disinfecting agents and act as reservoirs of histamine-producing bacteria [3]. The coating of intrinsic antibiofilm materials can reduce *L. parabuchneri* biofilm formation, with great industrial significance in the food-processing industry. Very few studies have been devoted to understand the antimicrobial activity of materials against *L. parabuchneri.* Recently, chitosan and its methylated derivatives have been reported to be active as antimicrobials to inhibit *Lentilactobacillus parabuchneri* biofilms [6]. In another study, an oregano-derived essential oil showed antimicrobial activity against *L. parabuchneri* [7].

The incorporation of metal and metal oxide nanoparticles into different support materials is a promising strategy to produce multifunctional nanocomposites for biosensors, catalysis, surface-enhanced Raman scattering, energy storage, and antimicrobial applications [8,9,10,11,12,13]. Among various materials, Ag-based nanocomposites have been widely investigated due to their unique properties and wide possibilities for application. The bioactivity of metal nanoparticles such as Cu, Zn, Au, Ti, and Ag is widely known. Among them, Ag NPs showed the most effective antimicrobial activity against a wide range of pathogenic bacteria, yeasts, fungi, and viruses [14]. Ag-based nanocomposites can exert multiple modes of action not only against sensitive bacterial strains, but also against highly resistant bacterial strains. The most commonly known mechanism of action disrupts bacterial metabolic processes, interacts with the DNA, and increases the cytoplasmic membrane permeability [15]. In particular, Ag-based composites were applied as antimicrobial agents in food packaging and biomedicine [16,17,18]. Various methods, from chemical to hybrid (physical and chemical) synthesis, have been developed to prepare AgNPs. Versatile laser ablation synthesis in solution (LASiS) allows for producing highly stable AgNPs using nontoxic chemicals [19] without requiring precursors and reducing agents. The green and simple LASiS technique produces NPs with unique surface characteristics and without any byproducts [20]. The preparation of NPs on solid support such as montmorillonite (MMT) is highly appreciated, as it can form practically applicable supported particles. MMT nanosheets allow for preparing multifunctional antimicrobial hybrid nanocomposites with peculiar mechanisms of action, and tunable and enhanced activity against antimicrobial resistant microorganisms. Moreover, the incorporation of colloidal NPs into an MMT matrix could lead to the perspective development of additives for real-life application. The MMT support substrate is highly attractive due to its excellent ion exchange capacity, chemical inertness, natural abundance, cost-effectiveness, nontoxicity, ease of exfoliation to two-dimensional nanosheets, and effortless dispersion in almost all solvents. Furthermore, MMT has the advantage of a lamellar structure and a large surface area, exhibits negative surface charge (−63 ± 11 mV), and is also biologically inert [21,22,23]. MMT/metal NP composites exhibit excellent adsorption, rheological, heat-insulator, heat-resistant, and swelling properties [24]. An appealing property of MMT substrates is the propensity to absorb a variety of compounds on their surface. The addition of beeswax into MMT-supported NPs could allow for the controlled release of antimicrobial ions. Beeswax contains more than 300 components: it is a mixture of hydrocarbons, free fatty acids, esters of fatty acids and fatty alcohol, diesters, and exogenous substances [25]. The diversity of this chemical composition gives an additional advantage as an antimicrobial agent, result in it being beneficial for antibiofilm application. The combination of many active ingredients and their presence in various proportions prevent bacterial resistance [26]. Beeswax possesses good antimicrobial activity against a wide range of pathogenic bacteria. Beeswax is commonly used in the cosmetic, food, and pharmaceutical sectors, including for the treatment of burns, wounds, bruises, and fractures [27]. It is considered to be one of the essential hydrophobic materials to reduce moisture permeability [28]. A beeswax surface coating results in a drastic decrease in water vapor penetration, whereas its effects on mechanical properties are generally irrelevant [29]. This property could be beneficial in controlling or slowing down the release of antimicrobial ions for long-term effects. The preparation and extensive analytical characterization of active NAMs play important roles in understanding their mechanism of action and overcoming toxicity issues. Long-term stability and cost-effectiveness can be improved in this way, too. Attenuated total reflection Fourier-transform infrared (ATR-FTIR) spectroscopy can be used for the in situ and real-time monitoring of early-stage biofilm formation with molecular specificity and metabolomic changes [30,31,32]. One of the major components in biofilms, the extracellular polymeric substance (EPS), plays an important role in biofilm formation, which can be monitored in real time [33].

In the present work, hybrid (physical and chemical) routes are adapted to prepare L-Ag/MMT composites with detailed analytical characterization. Laser-ablated Ag NPs were impregnated into refined MMT (L-Ag/MMT) with a simple wet chemical method using 2-propanol as dispersing solvent. Further, an L-Ag/MMT nanocomposite was introduced into beeswax to develop water-insoluble antimicrobial coatings. The inhibition of biofilm formation by the L-Ag/MMT/beeswax coating provides a strategy to reduce the contamination of food-processing equipment to prevent the spoilage of cheese due to histamine-producing species.

## 2. Results and Discussion

### 2.1. Characterization of L-Ag/MMT and L-Ag/MMT/Beeswax

**Figure 1:**
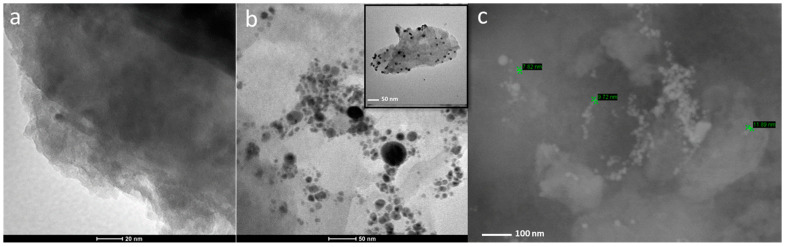
TEM images of (**a**) MMT and (**b**) L-Ag/MMT; SEM images of (**c**) L-Ag/MMT.

AgNPs were obtained with ns-pulsed laser ablation in pure isopropanol. The average colloidal concentration was equal to 0.08 ± 0.01 g/L. A TEM micrograph was acquired for L–Ag NPs. AgNPs showed a spherical morphology (Supplementary Materials Figure S1a–c) and an average size diameter of 10 ± 3 nm (Figure S1d). A typical TEM micrograph of the refined MMT and L-Ag/MMT nanocomposite can be seen in Figure 1a,b, respectively. TEM images confirmed the presence of spheroidal AgNPs entrapped into the surface layer of the MMT matrix. Ag NPs were well-dispersed on the MMT matrix without aggregation. A typical SEM image showed AgNPs on the surface of the MMT structures (Figure 1c).

**Figure 2:**
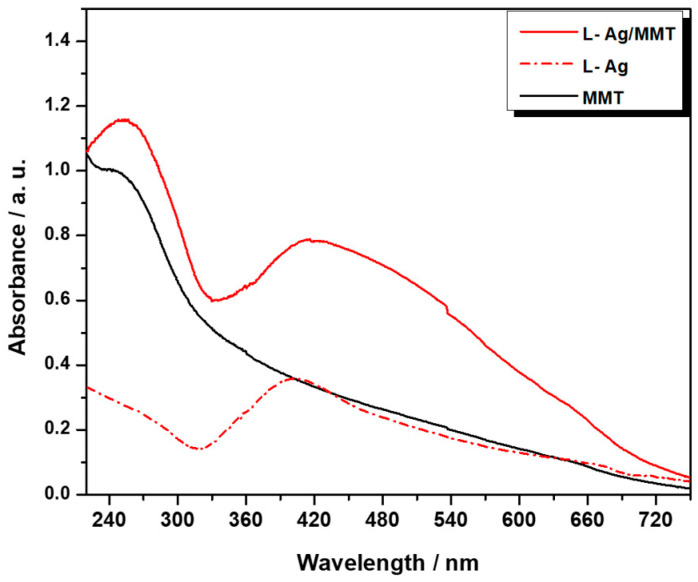
UV–vis absorption spectra of MMT, L-Ag, and L-Ag/MMT.

UV–vis spectra of MMT, L- AgNPs, and L-Ag/MMT can be seen in Figure 2. As expected, MMT displayed a characteristic broad band at 270–280 nm [34], L-Ag showed a surface plasmon resonance (SPR) peak at 400 ± 4 nm. In the case of L-Ag/MMT, the SPR of Ag NPs was observed at 418 ± 4 nm. L-Ag/MMT showed more intense absorption than that of bare MMT and L-AgNPs in the 240 to 450 nm range. Additionally, a shift towards higher wavelengths was observed that was probably associated with the plasmonic behavior of the interaction of small AgNPs with MMT particles [35,36,37,38].

**Figure 3:**
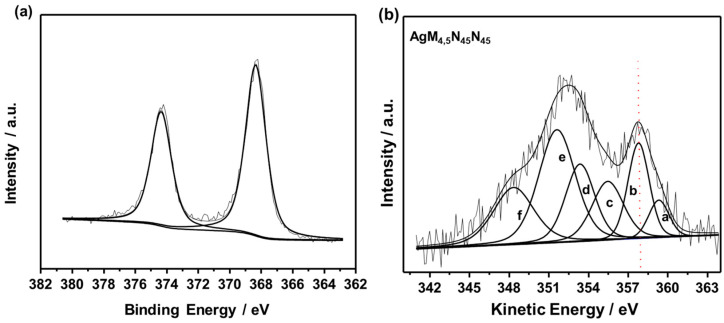
(**a**) Ag3d XP and (**b**) AgM_4,5_N_45_N_45_ Auger spectra of L-Ag/MMT.

XPS characterization was carried out to confirm the loading of L-Ag NPs into the MMT matrix. Table 1 presents the typical surface compositions of L-Ag NPs, L-Ag/MMT, and refined MMT samples, demonstrating that silver was detected in the L-Ag/MMT nanocomposite. The results of L-Ag NP characterization were in agreement with those in our previous studies, conducted on Ag NPs prepared via laser ablation in isopropanol, in terms of both elemental content and silver speciation [19]. The Ag surface composition was affected by the massive presence of organic matter coming from the degradation of IPA during LASiS, and this is not surprising on the basis of the literature [19]. Despite the very low amount of metal in the final composite, it had a very good antimicrobial and antibiofilm efficacy (vide infra). A more detailed analysis was performed on L-Ag/MMT samples to evaluate if any change in silver speciation had occurred in the nanocomposite. The Ag3d high-resolution region and AgMNN Auger spectrum are presented in Figure 3a,b, respectively. The complex Auger signal was curve-fit according to the literature; more details on the component attributions were reported by Bera et al. [39]. The Ag3d_5/2_ photoelectronic peak centered at 368.3 ± 0.2 eV (Figure 3a) was compatible with Ag(0) [40]. However, to corroborate this assumption, modified Auger parameter α’ was calculated by summing BEAg3d_5/2_ and KEAgM_4,5_N_45_N_45_ (357.8 eV, dashed red line in Figure 3b), giving a value of 726.1 ± 0.4 eV, which is undoubtedly ascribed to elemental silver in the MMT matrix [39].

**Figure 4:**
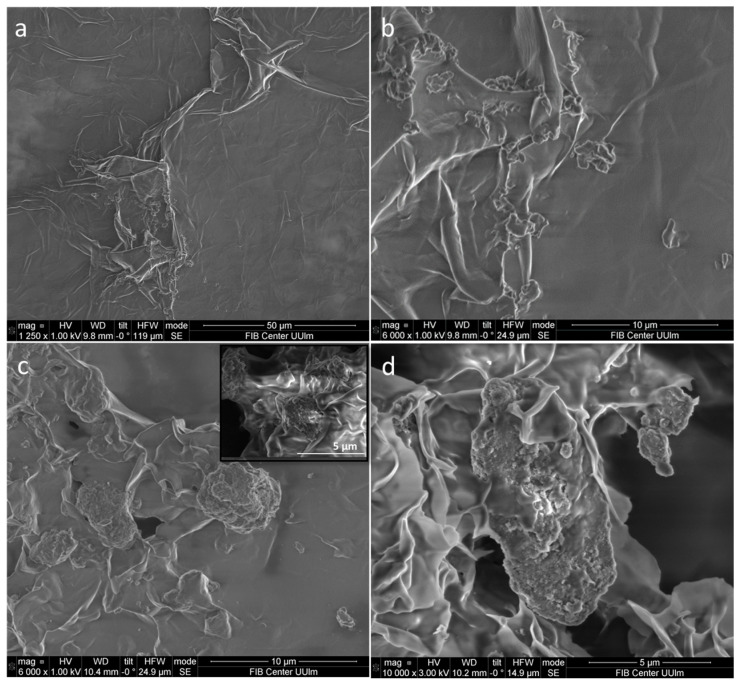
SEM images of (**a**,**b**) beeswax and (**c**,**d**) L-Ag/MMT/beeswax on silicon substrate.

The morphological characterization of beeswax and L-Ag/MMT/beeswax-coated silicon wafers was investigated with SEM. The surface of the silicon wafer was covered with a layer of beeswax film with a few wrinkles, as shown in Figure 4a,b. At higher SEM magnification in the L-Ag/MMT/beeswax sample, the beeswax structure was seen to hold MMT-supported Ag NPs (Figure 4c,d).

### 2.2. Kinetics of Silver Release

**Figure 5:**
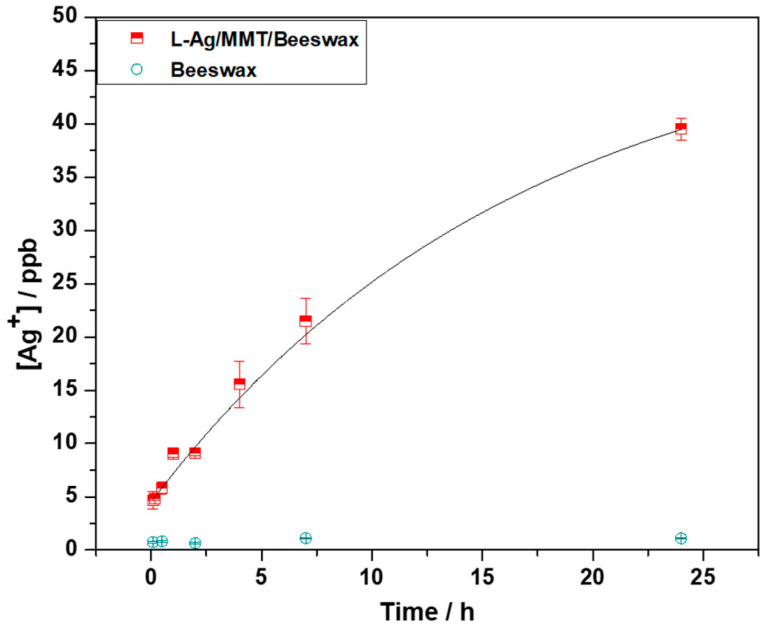
Ion release kinetics of L-Ag/MMT/beeswax coating.

The silver ion release kinetics was studied on Ag/MMT/beeswax. Experimental points were curve-fitted with a first-order kinetic curve with a rate constant equal to 17 ± 6 h^−1^. Ag^+^ concentration in the contact solution increased as a function of time, reaching a plateau at about 45 ± 9 ppb, as shown in Figure 5. These results indicate that there was a controlled release of Ag^+^ ions. The measured silver concentration is expected to exert long-term biostatic activity. In this context, a very low amount of Ag NPs in the final composite showed good antimicrobial and antibiofilm efficacy. This limited the final cost of the nanocomposite and reduced toxicological risks associated with the use of silver as bioactive material.

### 2.3. Biofilm Inhibition Efficiency on the Coated ZnSe Surface

Antiadhesive and antibiofilm L-Ag/MMT/beeswax nonantimicrobial coatings for eradicating these biofilms are based on the prevention of biofilm formation in quorum sensing (QS) inhibition via intercellular signaling mechanisms [41]. The biofilm inhibition pathway of AgNPs is attributed to interactions with the microbial membrane of bacteria and the disruption of membrane permeability [42]. AgNPs damage the bacterial outer membrane by inducing pits and gaps in the microbial membrane, and fragment the cell [43]. Microbial cells, in the outer surface of their membrane, carry negative charges resulting in electrostatic interaction between the antimicrobial L-Ag/MMT and cell membrane, and leading to the inhibition of *L. parabuchneri* growth. The prominent contribution is the incorporation of Ag into MMT as a solid supporting material for producing multifunctional hybrid nanocomposites, favoring strong ion exchange capacity between the nonantimicrobial and bacterial communities of cells. The ability of MMT to absorb compounds on its surface allows for its hydrophobic region and protective properties to merge of beeswax [44]. An investigation of the inhibitory mechanism via in situ FTIR-ATR spectroscopy provides a clear overview of the antibiofilm action of L-Ag/MMT/beeswax nanocomposite.

The nondestructive FTIR-ATR spectroscopy technique allows for a more comprehensive characterization of microbial biofilms in order to understand the fundamental mechanisms involved in bacterial adhesion and attachment [45]. Despite the complexity of IR spectra, it is evident that the contribution of bacterial biofilm components corresponds to stretching and bending vibrations including symmetric and asymmetric modes, respectively [46]. The approach used here allows for the in situ monitoring of *L. parabuchneri* biofilm formation and inhibition [47].

**Figure 6:**
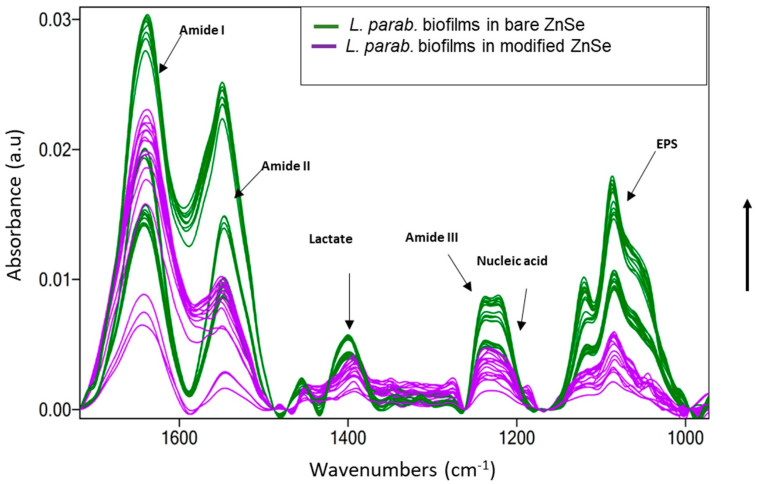
Infrared ATR spectra of 32 h old *L. parabuchneri* biofilms assigned by fluctuations in amides I–III, lactate, nucleic acid, and EPS as time-varying absorbance bands monitored (green line) on a bare ZnSe crystal and (purple line) on an L-Ag/MMT/beeswax modified ZnSe crystal at inactive sensing regions. The arrow in the y axis indicates the time evolution from 0 h (at the bottom) until 32 h (at the top).

On the bare surface of a ZnSe crystal, the initial biofilm attachment was manifested with a constant increase in the bands attributable to biofilm constituents. It was instantly seen that all bands related to amides I and II, nucleic acids, and extracellular polymeric substance (EPS) increased significantly over time. The constantly low increment of amide III and nucleic acid level indicates the amide bond in membrane proteins, fatty acid chains, and ribosomes as bacterial cell components assigned to these prominent band vibrations [48]. Figure 6 shows the spectra of *L. parabuchneri* biofilms on bare and L-Ag/MMT/beeswax-modified waveguides. During 32 h of monitoring on the bare crystal, the bands related to biofilms significantly increased over time, indicating the continuous coverage of the crystal surface with microbial biomass. In the case of the patterned crystal, the intensity of all the bands related to biofilms (amides I–III, lactate, nucleic acid, EPS) [49] slightly decreased over time. The stress generated from the compact and homogeneous nonantimicrobial coating slowed down biofilm proliferation. Particularly, the bands associated with amide I (1700–1616 cm^−1^), amide II (1578–1476 cm^−1^), lactate production (1465–1293 cm^−1^), amide III (1350–1200 cm^−1^), nucleic acid (1280–1175 cm^−1^), and extracellular polymeric substance (EPS) (1038–989 cm^−1^) became visibly lower, indicating the constant detachment of microbial biomass on the surface of the ZnSe waveguide.

**Figure 7:**
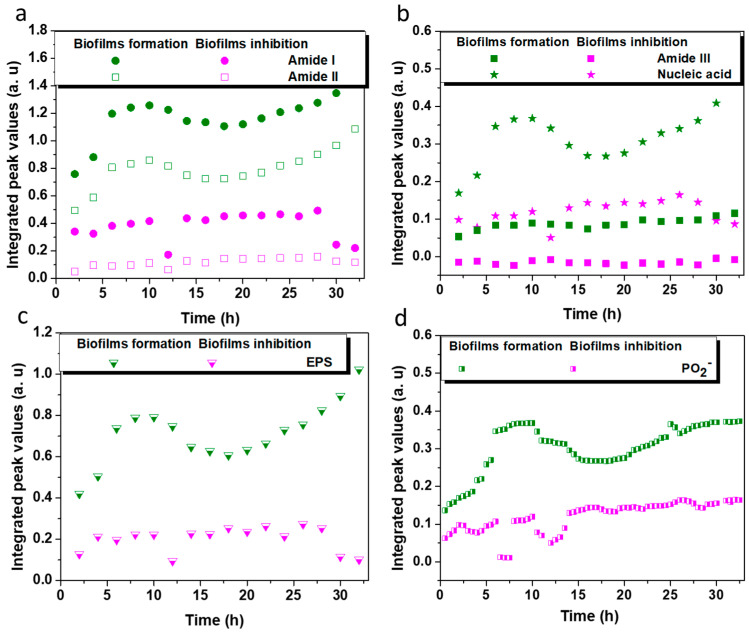
Integrated peak values (IPV) as a function of time for the *L. parabuchneri* biofilm formation on bare crystal (olive line) and biofilm inhibition on top of L-Ag/MMT/beeswax modified crystal (magenta line) for (**a**) amides I and II, (**b**) amide III and nucleic acid, (**c**) EPS matrices, and (**d**) ν_as_ PO_2_^−^ band obtained with in situ ATR-FTIR spectroscopy.

In Figure 7a, the integrated areas of the amide I and II bands are depicted for biofilms formed over a period of 32 h. It is immediately evident that a recurrent change in absorbance intensity was observed as a consistent decrease over hours of monitoring the flow system of the modified waveguide. This inhibition ability is indicative of the biostatic action of the L-Ag/MMT/beeswax composite film despite the tendency to reach zero. The amide I band, expressed in the infrared vibrations of υC=O coupled with N-H δH_2_O, was related to cell membrane constituents [50]. The amide II band, assigned to δN-H coupled with C-N vibrations, indicates proteins within the cytoplasm, flagella, pili, and membranes. Silver ions released by the L-Ag/MMT/beeswax coating could penetrate the cell membrane and interact with cell organelles, consequently leading to the disorganization of cell functionality [51]. This is the main reason for the constant decrement of absorbance intensity observed for amide I, and the same continuous decrease in amide II with much higher efficiency. It is apparent that there was less biofilm attached to the crystal surface. *Lentilactobacillus parabuchneri* is responsible for the contamination and food spoilage of cheese and other dairy products [52,53,54]. Being a basic biogenic amino compound with a flexible ethylamine side-chain moiety, histamine is released during the biofilm formation of *L. parabuchneri* [4], which is why controlling the amide range is important for tracing the histamine-producing biofilms of this specific bacterium [4,55]. The integrated peak values of amide III and nucleic acid shown in Figure 7b confirmed the gradual decrease in both nucleoid cell components after the direct contact of the bacterial cells with the antimicrobial coating. The different secondary protein structures of cell membranes and nucleoids reflect a greater spectral difference in the amide III range than that in amide I [56].

Extracellular polymeric substances (EPSs) are major biomolecules in microbial aggregates kept together in a three-dimensional matrix [57]. EPS are the main indicator of biofilm formation. Polysaccharides, phospholipids, phosphodiesters, etc. are the fraction of the EPS matrix that maintains the multicellular microbial communities [58,59]. The combination of bands revealing the EPS presence of biofilm matrix [30] consists of υC-O-C, υC-O, υC-C, δC-O-C, υsC-OH, υC-O, υsC-O-C, vibrational groups (1138–989 cm^−1^). The evaluation of EPS bands as a function of time for a bare and modified crystal is shown in Figure 7c. The intensity of the EPS band increased during *L. parabuchneri* biofilm formation on the bare crystal, reaching the maximum in about 8 h. EPS traces confirmed lactobacillus biofilm growth within the first hours of monitoring, similarly to any type of bacteria [60]. The nutrient gradient was increased over time with the switched inoculum into the FTIR-ATR flow cell, making space for a slight increase in the EPS band, while proteins in the bottom layer of biofilms spread horizontally [61]. In the case of the inactive spot experiment, the biofilms formed during exposure to L-Ag/MMT/beeswax coating, and a slower crystal coverage occurred. This is associated with the fact that less biofilm was attached to the surface due to the antimicrobial L-Ag/MMT/beeswax film. On that basis, all molecular bands after 2 h of monitoring reached almost zero, indicating an early stage of biofilm formation, except for amide III band, which was below zero. The efficient antibiofilm activity was related to biofilm death and the lysis process [62]. The antibacterial action was investigated through the evaluation of nucleic acid phosphate groups (~1220 cm^−1^) among other bands, related primarily to the metabolic activity of bacteria inside the biofilm [63,64]. The increment of asymmetric stretching PO_2_^−^ implied enhanced metabolomic cell activity of the *L. parabuchneri* biofilms close to the ZnSe waveguide surface (Figure 7d). The meager shift at 15–20 h of monitoring was attributed to the loss of proteins due to a lack of nutrients until the continuous inoculation of fresh MRS media into the flow FTIR-ATR system [24]. Then, for the modified crystal by antimicrobial film, a uniform decrease appeared after 10 h due to the stress induced by the nanomaterial film, along with a slow decrease in the signals for the continuous 32 h of monitoring, indicating that no colonization occurred on the entire ZnSe surface.

The decrement in biofilm microbial density was also associated with the sharp decrease in the intensity of the carbohydrates, protein vibrations υC-O, υsC-OH (1124 cm^−1^), phosphodiesters, phospholipid band υsPO_2_^−^ (1086 cm^−1^) as a correlation of membrane cell wall, and the very important polysaccharide functional groups, υO-H coupled with δC-O and υsC-O-C, υsP-O-C (1038 cm^−1^) from peptidoglycan [65], capsule and cell walls as cellular components (Table S1). Fluctuations in the EPS and phosphate bands with a decreasing trend were assigned to reduced cell activity [66] due to the distraction mechanisms coming from the nano-anti-microbial coating. The crystal bacterial colonization was impeded by the silver ions in the flow cell that were released into the system of biofilm monitoring. Furthermore, this permitted the decoupling of antibacterial effects that had been caused by Ag^+^ from the cellular stress induced by the direct contact of Ag NPs and *L. parabuchneri* bacteria via the oxidation of surface silver atoms from the dissolved oxidants in the cytoplasm of bacterial cells [51]. Regardless of the high impact from the beeswax hydrophobicity that influenced the late microbial attachment, the main interaction mechanisms of biofilm growth control were determined via the stress coming from nonantimicrobial coating. The molecular mechanisms of the antimicrobial action associated with AgNP-based coatings are necessary for the rational design of AgNPs for food industry packing and pharmaceutical formulations.

### 2.4. Optical Density Measurement and Microscopy on L. parabuchneri Biofilms

The OD of the bacterial suspension was measured after treatment for 24 h using the developed coating (Figure S2). A substantial decrease in OD values was observed for the L-Ag/MMT/beeswax coating, which indicated the potential inhibition of biofilm growth on the coated surface. In the presence of the beeswax coatings used as controls, almost the same OD values as those of uncoated silicon wafer were observed.

**Figure 8:**
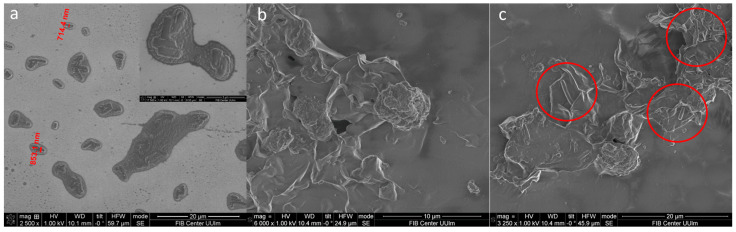
SEM images of (**a**) silicon substrate and (**b**,**c**) L-Ag/MMT/beeswax coating incubated in *L. parabuchneri* bacterial suspension for 24 h.

The silicon surface is easily colonized by bacterial cells. Hence, bacterial biofilm colonization was observed on the pristine silicon substrate and L-Ag/MMT/beeswax-coated silicon substrate via SEM analyses. Figure 8a shows *L. parabuchneri* after 24 h incubation on bare silicon, which clearly reveals that most of the bacterial cells were embedded in small colonies. The development of *L. parabuchneri* can be defined as structural organization showing a dense network of rod-shaped *L. parabuchneri* cells. The L-Ag/MMT/beeswax-modified surface significantly inhibited the formation of colonies, as can be seen in Figure 8b. The antimicrobial-coated surface could substantially reduce the cell metabolism rate, causing changes in the bacterial colony structure [67]. Further, antimicrobial influence could destroy the network of EPS matrices and disintegrate the biofilms into individual cells [68]. A very low number of individual cells were observed on the L-Ag/MMT/beeswax-coated surface, as shown in detail in Figure 8c. This could have been due to the intrinsic hydrophobic nature of the coating [28]. In addition, the intrinsic antimicrobial activity of silver ions and their controlled release could be what hindered the formation of bacterial communities.

## 3. Materials and Methods

### 3.1. Materials

Silver sheets (99.99% purity, diameter: 10 mm; nominal thickness: 1 mm) were purchased from Good Fellow Ltd. (Cambridge, UK). Isopropanol (IPA, >99.9%, HPLC grade), ethanol (EtOH, absolute, ACS reagent), HNO_3_ (67%, Trace-SELECT^®^ Ultra, for ultratrace analysis), NaCl (Trace-SELECT^®^ Ultra, ≥99.999%, for ultratrace analysis), Cu grids (Agar Scientific, Stansted, UK), carbon-coated, 300-mesh were used for the preparation of TEM samples. Montmorillonite (K10, powder) was bought from Sigma Aldrich, and beeswax (bleached, white) from Acros Organics.

### 3.2. Preparation of L-Ag NPs, L-Ag/MMT Nanocomposite, and L-Ag/MMT/Beeswax Coating

The synthetic steps from L-Ag NPs and L-Ag/MMT composite towards the final water-insoluble hard L-Ag/MMT/beeswax coating are depicted. First, L-Ag NPs were prepared following a previously published work [19]. In brief, the LASiS experimental setup consisted of a nanosecond Q-Switched Nd:YAG Laser (Quantel-BRIO) operating at a fundamental wavelength of 1064 nm with a pulse frequency of 20 Hz, a pulse energy of 46.5 mJ, and a nominal pulse duration of 4 ns. The Ag target, after mechanical polishing with wet SiC grinding paper and sonication for 15 min in IPA, was fixed to the wall of a 35 × 25 × 30 mm^3^ vessel filled with 10 mL of IPA. A yield of 0.08 mg/mL of Ag NPs in IPA was calculated for 20 min of ablation.

Following that, L-Ag/MMT was prepared by simply mixing L-Ag colloids and MMT. It was necessary to perform washing and refining steps to make the MMT matrix suitable for the accommodation of NPs into/onto its layer/surface. To this aim, MMT (K10) was dried overnight at 80 °C. After that, dried MMT was ground using a mortar and pestle, and washed in deionized water at a ratio of 50 mg MMT:1 mL water for 30 min. Larger particles were removed with sedimentation; the upper portion of the suspension was centrifuged, and subsequently dried and ground to obtain refined MMT. Refined MMT was dispersed in IPA and subsequently previously prepared L-AgNPs/IPA solution was added to the MMT/IPA dispersion at an Ag:MMT ratio of 1:37. The mixture was then stirred at room temperature for 60 min until well-dispersed MMT in IPA had been acquired. The mixture was lastly centrifuged at 5000 rpm for 5 min to collect the L-Ag/MMT composite. The sample was washed 3 times with 99.9% IPA.

L-Ag/MMT 0.5%*w*/*w* composites were mixed with 10 g/L beeswax in IPA under stirring at 60 °C for 10 min. Then, 500 μL of this suspension was deposited onto 2 × 2 cm^2^ glass slides, Si wafers, or aluminum foil via spin coating with a rotation program of 2500 rpm for 20 s.

### 3.3. TEM and SEM Morphological Characterization

TEM analysis was performed using an FEI Tecnai 12 instrument (120 kV; filament: LaB_6_). Samples were drop-cast onto carbon grids (300 mesh, Agar Scientific, Stansted, UK) at a volume of 2 to 3 μL for each sample. Size distribution analysis was performed with ImageJ software. The morphology of the L-Ag/MMT composite was evaluated with FE-SEM model Sigma Zeiss (Jena, Germany), 10 kV acceleration voltage, using a 30 nm aperture and inlens detector. The coatings before and after incubation with the bacteria were assessed via SEM measurements with a dual-beam FIB/SEM system (Quanta 3D FEG, FEI Company, Eindhoven, The Netherlands).

### 3.4. XPS and UV–Vis Characterization

L-Ag NPs and L-Ag/MMT composites were analyzed with a PHI 5000 Versa Probe II Scanning XPS Microprobe spectrometer (ULVAC-PHI Inc., Kanagawa, Japan). The measurements were performed using a monochromatized Al Kα source (X-ray spot 200 µm) at power of 50.3 W. Wide scans and detailed spectra were acquired in fixed analyzer transmission (FAT) mode with pass energies of 117.40 and 46.95 eV, respectively. An electron gun was used for charge compensation (1.0 V, 20.0 µA). The binding energy (BE) scale was calibrated by fixing the aliphatic component of the C1s signal (BE = 284.8 ± 0.1 eV) as the reference. Data processing was performed by using MultiPak software v. 9.9.0.8. The absorption spectra of L-Ag, L-Ag/MMT, and MMT were measured using UV–vis spectroscopy (Shimadzu UV-1601, Duisburg, Germany), operating the spectrometer in the 220 and 800 nm range in quartz Suprasil^®^ cuvettes from Hellma Analytics (Müllheim, Germany). A total of 3.5 mg/mL of L-Ag/MMT was used for the UV–vis experiment.

### 3.5. Determination of Silver Release

The kinetics of silver released from the L-Ag/MMT/beeswax coating into aqueous contact solutions was determined through ETAAS. Glass slides were laid onto a small Petri dish and covered with 3 mL of the contact solution. The contact solution was a 1:1 mixture of phosphate buffer (pH 6.8, ionic strength 0.1) and 0.85% *w*/*w* NaCl. Contact solution aliquots were mineralized with 0.5% HNO_3_ and then analyzed. The release experiments were conducted at a controlled temperature (21 ± 2 °C) in an air-conditioned room and at controlled environmental humidity (75 ± 2%), thus mimicking real-life conditions.

### 3.6. Bacterial Growth and Culture Conditions

The DSMZ 5987 strain of lactic acid bacterium *Lentilactobacillus parabuchneri* was obtained from the Leibniz Institute, German Collection of Microorganisms and Cell Cultures (DSMZ), Niedersachsen, Germany. The bacterial cells were maintained in Man de Rogosa Sharpe (MRS) broth in a microaerophilic environment during incubation at 37 °C for 24 h. At the end of the exponential growth phase, the optical density (OD-600) of the bacterial solutions was monitored using a UV–vis spectrophotometer (Specord S600, Analytik Jena AG, Germany). Cells were resuspended in fresh MRS medium to reach OD600 = 0.7 ready for in situ IR spectroscopy. The strain was isolated at −80 °C in MRS with 10% (*w*/*v*) sterile glycerol.

### 3.7. Modification of ZnSe Waveguide in Inactive Spots

FTIR-ATR spectroscopy was used to evaluate the biofilm inhibition ability of the L-Ag/MMT/beeswax coating. To conduct this experiment, we determined the evanescent wave regions along the surface of a 72 × 10 × 6 mm trapezoidal horizontal ATR crystal (45° in coupling facet, six reflections) produced from ZnSe. The crystal was mounted onto a customized horizontal flow cell with the top plate removed, facilitating the deposition of absorbing blended materials droplets at the crystal surface. The ZnSe crystal was modified in a way that only IR inactive regions of the waveguide were covered by the deposited L-Ag/MMT/beeswax blend. This approach allowed for simplifying the system, limiting bioactivity mechanisms only to the biofilm exposure towards metal ions. Previous studies showed the localization of IR-active sensing regions [64,69], helping to determine IR-inactive areas along the crystal surface; thus, we avoid infrared signals being convoluted with absorption intensity that might come from the composite film. The active spots were able to be attracted by the sampled biofilms as a consequence of exposure only to the Ag^+^ ions released by the neighboring regions (3 mm spots of nanoantimicrobials, 0.5 mm thickness). In this regard, two experiments were carried out: (1) modified ZnSe areas in the inactive sensing regions exactly between the internal reflections of the propagating IR radiation, and (2) the bare ZnSe crystal without any pattern on top of it for the normal in situ monitoring of *Lentilactobacillus parabuchneri* biofilm formation.

### 3.8. In Situ FTIR-ATR Spectroscopic Strategy as a Biofilm Inhibition Study

The flow system assembly designed for real-time IR measurements consisted of a flow cell setup connected to the FTIR-ATR multireflection compartment of the Tensor II infrared spectrometer (Bruker Optics, Etlingen, Germany) and a peristaltic pump (Watson Marlow Series 400, Cornwall, UK) with silicon tubing. The spectrophotometer was equipped with a DTGS detector and a six-reflection ZnSe crystal. The flow cell was mounted into the sample chamber of the infrared spectrometer. Prior to the in situ measurements, the crystal was cleaned in a UV light chamber using intense ultraviolet light. Afterwards, the mounted cell was cleaned with ethanol and rinsed with sterilized water for half an hour. The MRS conditioning film was recorded as a background spectrum for the subsequent *L. parabuchneri* biofilms to minimize water interference [30]. The sterilized MRS medium was flushed in the system for 3 h with a delay of 5 min between measurements at a flow rate of 0.7 mL/min, which resulted in a residence time within the flow cell of ~150 s. After recording the conditioning film background, the MRS media were replaced with a bacterial solution (OD-600 = 0.7) for 2 h at a 0.7 mL/min flow rate with 10 min delay between measurements. This period was optimized on the basis of the required time for initiating the attachment of *Lentilactobacillus parabuchneri* biofilms at the ZnSe waveguide. After 2 h of initial attachment of *L. parabuchneri*, a sterile MRS medium (52 g/L) was pumped again through the FTIR-ATR assembly cell for 20–24 h at a 0.5 mL/min flow rate. A fresh MRS solution medium washed out the free cells that had not been attached and delivered nutrients for the microorganisms. The spectra were recorded at 2 cm^−1^ resolution with 100 averaged spectra and in the infrared region of 4000–400 cm^−1^ (1700–989 cm^−1^ region of interest). Data acquisition was realized with Bruker OPUS™ 8.1 software. Spectra were all converted into Excel datasets using Essential FTIR software (Operant LLC, Madison, WI, USA), and elaborated in the OriginLab software package (OriginLab Corp., Northampton, MA, USA). The IR spectra of the *L. parabuchneri* biofilms were recorded at 22 ± 1 °C in an air-conditioned room. The plotting of integrated peak values (IPVs) was calculated from FTIR on the basis of six spectral regions: amide I (1700–1616 cm^−1^), amide II (1578–1476 cm^−1^), amide III (1350–1200 cm^−1^), nucleic acid (1280–1175 cm^−1^), EPS (1138–989 cm^−1^), and ν_as_ PO_2_^−^ band at 1086 cm^−1^ vs. time. The observed bands associated with the molecular components related to the *L. parabuchneri* biofilms showed the progression of the IR signature, which frequently changed as a function of time [70].

### 3.9. Biofilms Grown on Silicon Wafer

We immersed 1 × 1 cm^2^ coated silicon wafers in 15 mL of MRS and 150 µL of bacterial suspension (OD = 0.7), and they were incubated for 24 h at 37 °C. Following that, staining was fixed in 2.5% glutaraldehyde and dehydrated with a grated series of acetone solutions (50–100%). After drying, the samples were subjected to SEM analyses.

## 4. Conclusions

In this contribution, a water-insoluble L-Ag/MMT/beeswax antibiofilm coating was developed. MMT was used to support the laser-ablated AgNPs; L-Ag/MMT was then dispersed into beeswax for the controlled release of antimicrobial ions. A microscopic study confirmed the decoration of MMT with spherical AgNPs embedded into beeswax films. UV–vis spectroscopy indicated evidence of the complexation of metal AgNPs with the MMT supporter. XPS characterization provided a detailed surface chemical speciation of the L-Ag/MMT composite, including information regarding Ag chemical speciation, which was revealed to be Ag(0). A silver ion release investigation allowed for us to hypothesize that the L-Ag/MMT/beeswax coating could slow down biofilm formation while exerting mild antimicrobial action. The antibiofilm action was tested against the histamine-producing *Lentilactobacillus parabuchneri* bacterium. The developed coating is expected to inhibit biofilm formation. The IR -ATR flow analytical technique was used to monitor in situ the inhibition of the *L. parabuchneri* biofilm for 32 h, providing molecular insight into this phenomenon. The results indicate that the L-Ag/MMT/beeswax coating was able to slow down biofilm formation. This study indicated for the first time the in situ molecular changes of *L. parabuchneri* biofilm formation close to real time via flow FTIR-ATR spectroscopy under the influence of an L-Ag/MMT/beeswax coating. Furthermore, OD measurements confirmed the antimicrobial activity of this coating,. Last, the evaluation of the antibiofilm activity was supported with an SEM investigation on the bacteria incubated in contact with the antimicrobial coating.

## Figures and Tables

**Table 1:** Typical surface chemical composition for L-Ag NPs, L-Ag/MMT, and MMT. Errors are expressed as standard deviation on three analysis points.

Samples	At%								
	C	O	Na	Mg	Al	Si	Fe	S	Ag
L-Ag	92.6 ± 0.8	7.1 ± 0.7	/	/	/	/	/	/	0.3 ± 0.1
L-Ag/MMT	16 ± 5	55 ± 7	0.4 ± 0.2	1.0 ± 0.2	7.8 ± 1.8	17.7 ± 2.2	0.5 ± 0.2	0.5 ± 0.2	1.1 ± 0.3
MMT	26.4 ± 1.6	52.6 ± 0.6	/	0.6 ± 0.1	3.0 ± 0.2	17.0 ± 0.7	0.4 ± 0.2	/	/

## Data Availability

Not applicable.

## Supplementary Materials

The following supporting information can be downloaded at: https://www.mdpi.com/article/10.3390/antibiotics12020194/s1. Figure S1: (a–c) TEM images and (d) histogram of L-AgNPs; Table S1: infrared vibrational band assignment of the *Lactobacillus parabuchneri* cell and biofilm functional groups; Figure S2: optical density (OD) measurement of *L. parabuchneri* bacterial suspension after 24 h incubation treated with L-Ag/MMT/beeswax, beeswax coatings, and without coating (bare silicon wafer). References [45,50,65,66,71,72,73] are cited in the supplementary materials.
