# Supplementary material for: Preparation of Laser-Ablated Ag Nanoparticle–MMT Clay-Based Beeswax Antibiofilm Coating
Source: Antibiotics (Basel). 2023 Jan 17;12(2):194. doi: 10.3390/antibiotics12020194 (PMC9952241; doi:10.3390/antibiotics12020194)
Supplement: Supplementary file 1 [file antibiotics-12-00194-s001.zip › antibiotics-2168023-supplementary.pdf]

Supporting Information (SI)

Preparation of laser ablated Ag nanoparticles-MMT clay based Beeswax antibiofilm coating

Syed Imdadul Hossain[1,2], Diellza Bajrami[3], Maria Chiara Sportelli[1], Rosaria Anna Picca[1,2], Annalisa Volpe[4], Caterina Gaudiuso[4], Antonio Ancona[4], Luigi Gentile[1,2], Gerardo Palazzo[1,2], Nicoletta Ditaranto[1,2], Boris Mizaikoff[3,5], Nicola Cioffi[1,2*]

[1]*Chemistry Department, University of Bari "Aldo Moro", via E. Orabona 4 − 70126 Bari, Italy*

[2]*CSGI (Center for Colloid and Surface Science) c/o Dept. Chemistry, via Orabona 4, 70125 Bari, Italy*

[3] *Institute of Analytical and Bioanalytical Chemistry, Ulm University, Albert Einstein-Allee 11, 89081 Ulm, Germany*

[4] *IFN-CNR, Physics Department "M. Merlin", via Amendola 173, 70126, Bari, Italy*

[5] *Hahn-Schickard, Institute for Microanalysis Systems, Sedanstrasse 14, 89077, Ulm, Germany*

Email: nicola.cioffi@uniba.it

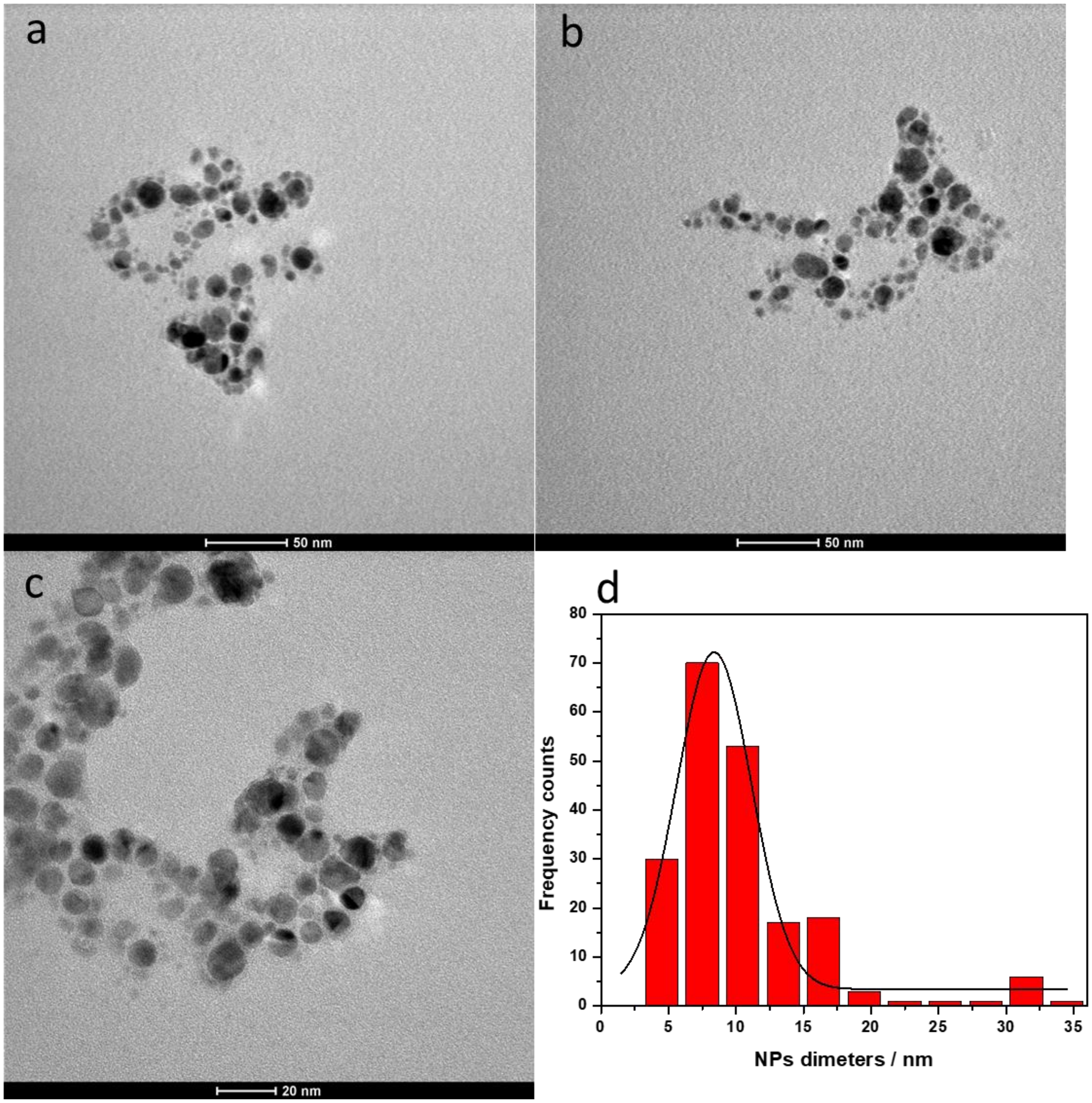

Figure S1. (a-c) TEM images and (d) Histogram of L-AgNPs

Table S1. Infrared vibrational band assignment of the *Lactobacillus parabuchneri* cell and biofilm functional groups.

Wavenumber (cm^{-1})	Functional group assignment
1700-1616	Stretching C=O coupled with N-H δH2O from membrane lipids and fatty acids (amide I band) [S1]
1578-1476	δN-H bending and C-N stretching in proteins (amide II band) [S2]
1468-1455	δCH2, δaCH3 bending from membrane lipids [S3]
1465-1293	υsCOO- of lactic acid and fatty acid chains [S4]
1350-1200	τCH2, ρCH2, υC-N coupled with δN-H (amide III band) [S5]
1280-1175	vibrations of -COOH and C-O-H; Double bond stretching >P=O of general phosphoryl groups and phosphodiester of nucleic acids [S6]
1225	Stretching of P=O in phosphates [S5]
1124	Symmetric stretching υsC-OH, υC-O of carbohydrates [S3]
1086	Stretching of P=O in phosphates, υsPO^{2-} [S7]
1038-989	Stretching of P=O of phosphodiester, phosphorylated proteins, phospholipids and polyphosphate products [S5]
976	Symmetric stretching vibration of phosphoryl groups (υC- C, υP-O-P) [S3]

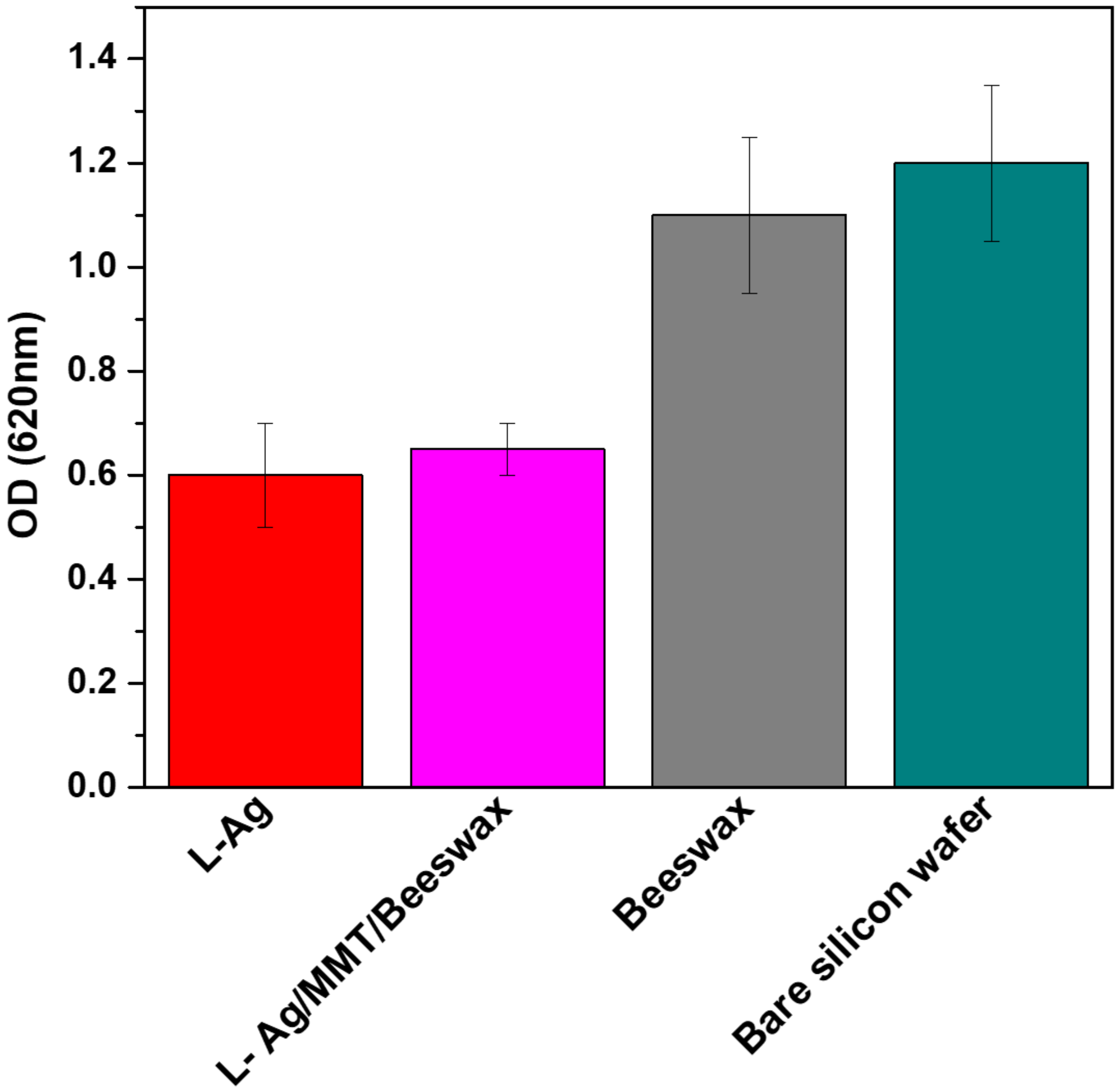

Figure S2. Optical density (OD) measurement of *L. parabuchneri* bacterial suspension after 24 h incubation treated with L-Ag/MMT/Beeswax, Beeswax coatings, and without coating (Bare silicon wafer)

REFERENCES

[S1] Schmitt, J.; Fringeli, U.P.; Flemming, H.-C. Structural and Temporal Behavior of Biofilms Investigated by FTIR-ATR Spectroscopy. **2010**, 312–315, doi:10.1063/1.55788.

[S2] Sharma, G.; Prakash, A. Combined Use of Fourier Transform Infrared and Raman Spectroscopy To Study Planktonic and Biofilm Cells of Cronobacter Sakazakii. *J Microbiol Biotechnol Food Sci* **2014**, *9*, 310–314.

[S3] Ojeda, J.J.; Dittrich, M. Fourier Transform Infrared Spectroscopy for Molecular Analysis of Microbial Cells. *Methods Mol Biol* **2012**, *881*, 187–211, doi:10.1007/978-1-61779-827-6_8.

[S4] Oust, A.; Møretrø, T.; Kirschner, C.; Narvhus, J.A.; Kohler, A. FT-IR Spectroscopy for Identification of Closely Related Lactobacilli. *J Microbiol Methods* **2004**, *59*, 149–162, doi:10.1016/j.mimet.2004.06.011.

[S5] Ojeda, J.J.; Romero-Gonzalez, M.E.; Pouran, H.M.; Banwart, S.A. In Situ Monitoring of the Biofilm Formation of Pseudomonas Putida on Hematite Using Flow-Cell ATR-FTIR Spectroscopy to Investigate the Formation of Inner-Sphere Bonds between the Bacteria and the Mineral . *Mineral Mag* **2008**, *72*, 101–106, doi:10.1180/minmag.2008.072.1.101.

[S6] Sportelli, M.C.; Tütüncü, E.; Picca, R.A.; Valentini, M.; Valentini, A.; Kranz, C.; Mizaikoff, B.; Barth, H.; Cioffi, N. Inhibiting P. Fluorescens Biofilms with Fluoropolymer-Embedded Silver Nanoparticles: An in-Situ Spectroscopic Study. *Sci Rep* **2017**, *7*, doi:10.1038/s41598-017-12088-x.

[S7] McWhirter, M.J.; Bremer, P.J.; McQuillan, A.J. Direct Infrared Spectroscopic Evidence of PH- and Ionic Strength-Induced Changes in Distance of Attached Pseudomonas Aeruginosa from ZnSe Surfaces. *Langmuir* **2002**, *18*, 1904–1907, doi:10.1021/la010928k.
